# Chronic intermittent exposure to ayahuasca during aging does not affect memory in mice

**DOI:** 10.1590/1414-431X20176037

**Published:** 2017-06-05

**Authors:** N.F. Correa-Netto, L.S. Coelho, G.S. Galfano, F. Nishide, F. Tamura, M.K. Shimizu, J.G. Santos, A. Linardi

**Affiliations:** Departamento de Ciências Fisiológicas, Faculdade de Ciências Médicas, Santa Casa de São Paulo, São Paulo, SP, Brasil

**Keywords:** Anxiety, Banisteriopsis, Carbolines, N,N-dimethyltryptamine, Learning

## Abstract

The Quechua term ayahuasca refers to a beverage obtained from decoctions of the liana *Banisteriopsis caapi* with leaves of *Psychotria viridis*. The ritualistic use of ayahuasca is becoming a global phenomenon, with some individuals using this beverage throughout life, including in old age. Cognitive impairment is a common manifestation during aging. There are conflicting reports on the ability of some ayahuasca compounds to exert neuroprotective or neurotoxic effects that could improve or impair learning and memory. Animal models provide a relevant and accessible means of investigating the behavioral effects of ayahuasca without the environmental conditions associated with the ritualistic use of the beverage. In this study, we investigated the influence of chronic ayahuasca exposure throughout aging on the spatial reference and habituation memories of mice. Twenty-eight male c57bl/6 mice (6 months old) received ayahuasca or water (1.5 mL/kg, orally) twice a week for 12 months and were tested in the Morris water maze (MWM), open field and elevated plus maze (EPM) tasks before and after treatment. During aging, there was significant impairment in the evocation (but not acquisition) of spatial reference memory and in habituation to the open field. There was also a decrease in locomotor activity in the open field and EPM tests, whereas the anxiety parameters were unaltered. Ayahuasca treatment did not alter any of these parameters associated with aging. These findings indicate that chronic exposure to ayahuasca during aging did not affect memory in mice.

## Introduction

The Quechua term ayahuasca, common in Peru, Bolivia, Brazil and parts of Ecuador, is used to refer to decoctions of the liana *Banisteriopsis caapi* with the leaves of *Psychotria viridis.* The result of decoction is a thick, brown liquid (1). *Banisteriopsis caapi* contains the alkaloids harmine, harmaline and tetrahydroharmine, all of which belong to the β-carboline group of compounds. The leaves of *P. viridis* contain the alkaloid N,N-dimethyltryptamine (DMT), the main substance responsible for the hallucinogenic effects of ayahuasca (2).

DMT is structurally similar to serotonin and its central effects result from agonistic activity at serotonergic 5-HT_1A_, 5-HT_2A_ and 5-HT_2C_ receptors (3,4). Harmine, tetrahydroharmine and harmaline are potent inhibitors of peripheral type-A monoamine oxidase (MAO-A). MAO inhibition in the gastrointestinal tract increases DMT bioavailability in the central nervous system, and these alkaloids play a pivotal role in the hallucinogenic effects of DMT (2). The hallucinogenic experiences induced by ayahuasca include modifications in perception, mental processes and recollection of recent events with a marked emotional content. In addition, transient nausea, vomiting and episodes of diarrhea can also occur after ayahuasca consumption (2). The psychotropic effects start 35–40 min after oral ingestion, reach maximal intensity after 90–120 min and end after 4 h (2,5).

There are several ancient indigenous and mestizo traditions that use ayahuasca, whereas the emergence of its organized, urban, non-indigenous and religion-based use in Brazil is a recent phenomenon (6). The most commonly recognized sacramental use of ayahuasca occurs among members of three churches in Brazil: União do Vegetal (UDV), Santo Daime and Barquinha. Since ayahuasca use in these religions is legally permitted in Brazil as part of the freedom of religious worship guaranteed by the country's Constitution, the control of ayahuasca use is affected primarily through regulatory procedures contained in Resolution No. 5 (November 4, 2004) that culminated in the publication of Resolution No. 1, on January 25, 2010 by the Conselho Nacional de Políticas sobre Drogas (CONAD, or National Council on Drug Policies) (7).

In addition to its religious use, there is evidence for a potential therapeutic effect of ayahuasca. Two independent studies found that ayahuasca users in religious groups had lower scores of anxiety, panic and hopelessness compared to participants receiving placebo (5,8). An important question relates to the safety of ayahuasca in regular consumers of the decoction. Based on the assessment of psychological parameters such as cognitive functions, verbal fluency, math ability, motivation, emotional well-being and personality traits, Grob et al. (2) found no evidence of mental impairment in 26- to 48-year-old chronic ayahuasca users. More recently, a review of the emotional, cognitive and physical health of ayahuasca users showed that it was safe and may be beneficial under certain conditions (9). In addition, studies *in vitro* have shown that β-carbolines, including harmine, have antimutagenic, antigenotoxic and antioxidant effects (10).

A decline in learning and memory is a common consequence of biological aging (11,12). This impairment is partly attributable to oxidative damage, mainly in the hippocampus (13). Since most members of ayahuasca churches use this beverage throughout their life and into old age, this ritualistic consumption of ayahuasca could potentially minimize the natural cognitive deficits seen during aging. This is an important issue to address given the reported therapeutic potential of ayahuasca. For this reason, in this study we investigated the effects of chronic ayahuasca exposure on learning and memory impairment during aging in mice. In order to mimic the frequency of ayahuasca ingestion in ritualistic use (the decoction is not ingested daily), we used a protocol of intermittent exposure (twice a week) to ayahuasca.

## Material and Methods

### Subjects

Twenty-eight male c57bl/6 mice obtained from the Center for the Development of Animal Models in Biology and Medicine at the Universidade Federal de São Paulo (CEDEME/UNIFESP, São Paulo, SP, Brazil) were used. The mice were housed 4–6 per propylene cage (40×34×17 cm) with woodchip bedding, at 20–22°C and 50% humidity on a 12-h light/dark cycle (lights on at 7:00 am), with free access to mouse chow pellets and tap water. The experimental protocols were approved by an institutional Committee for Ethics in Animal Use (CEUA/ISMSCSP, protocol No. 002/14) and the general ethical guidelines for animal use established by the Brazilian Society of Laboratory Animal Science (SBCAL), Brazilian legislation (Federal law No. 11,794, of October 8, 2008) and EU Directive 2010/63/EU were followed, in conjunction with the guidelines for animal experiments established by the Brazilian National Council for Animal Experimentation (CONCEA, http://www.mct.gov.br/index.php/content/view/310553.html; http://www.mct.gov.br/upd_blob/0234/234054.pdf). The mice were 6 months old at the start of the experiments.

### Ayahuasca

Ayahuasca was kindly provided by the Centro de Desenvolvimento Integrado Luz do Vegetal (Araçariguama, SP, Brazil). To prepare the beverage, the liana *B*. *caapi* was carefully washed in water and pounded with wooden mallets, whereas the leaves of *P*. *viridis* were simply rinsed with water. The plant material was boiled and concentrated over several hours to produce approximately 100 L of beverage. The ayahuasca extract used in the present study was the same used in a previous study by our group (14). The concentrations of psychoactive alkaloids detected by HPLC-DAD were 2070 µg/mL N,N-dimethyltryptamine, 147.5 µg/mL harmaline, 2894 µg/mL harmine, and 1893 µg/mL tetrahydroharmine.

### Treatment

Ayahuasca was administered orally at a dose of 1.5 mL/kg. This dose was based on the volume used by humans in religious rituals, which is approximately 100 mL/70 kg (15) and was also considered a typical dose in a previous study in Wistar rats (16). The stock solution of ayahuasca was diluted to facilitate oral administration. The mice (n=13) received the beverage twice a week by gavage since ayahuasca use in religious rituals does not occur daily, but intermittently. The control group (n=9) received water in a similar procedure. The treatment began when the mice were 6 months old and ended 12 months later, when the mice were 18 months old. Over this period, each mouse received 100 oral administrations. Six mice (5 in the control group and 1 in the ayahuasca group) died during the treatment period. Weight loss was used as an indicator of possible toxicity of the chronic intermittent exposure to ayahuasca so the mice were weighed once a week.

### Behavioral tests

All behavioral tests were done between 10:00 am and 3:00 pm in a sound-proof room, before treatment with ayahuasca and 24 h after the last administration of beverage at the end of the 12-month period. The tests were run for 7 days (D1, D2, D3, D4, D5, D6, and D7) before and after treatment. The open field and elevated plus maze (EPM) tests were run on D1 (in the morning and afternoon, respectively) and the Morris water maze (MWM) test was done from D2 to D7 (in the morning). The performances of the mice were recorded in all tests and the data were analyzed by an image capture system (SMART 2.1, Panlab, Spain).

### Open field test

The open field test was used to assess locomotor activity, an important indicator of drug toxicity, and also to rule out any confounding effect on the learning and memory test (MWM). Furthermore, locomotor activity was analyzed in five blocks of 5 min each, which allowed us to determine habituation, one of the most common forms of non-associative memory seen in rodents (17). The mice were placed individually in an acrylic cage (23×42×30 cm) and the distance covered was measured in 5-min blocks over 25 min.

### EPM test

The EPM test is a paradigm for assessing anxiety since it is based on the natural aversion of rodents to open and elevated areas, with these animals generally preferring dark, closed areas. Hence, the relative exploration in the open arms compared to both open and closed arms indicates the level of anxiety (18). The apparatus consists of four arms (30×5 cm) elevated 40 cm above floor level and connected to each other by a central platform (5×5 cm). Two of the arms are closed with 18-cm-high walls and the other two arms are open. Each mouse was placed in the center section and left to explore the maze for a single 5-min session. The number of entries and time spent in the open and closed arms were measured. These variables allowed us to determine the percentage of exploration (entries and time spent) in the open arms in relation to both open and closed arms. The number of entries into the closed arms was also used as an indicator of general locomotor activity.

Several studies have suggested that more detailed behavioral analyses improve the validity of the EPM test (18,19). To complement our findings, we measured the time spent in risk assessment, a stretched-attend posture directed to both open and enclosed arms, in which the animal demonstrates forward elongation of the neck and shoulders followed by retraction to the original position (18,20). The risk assessment behavior was estimated by observing the movement of the mouse from the closed arms to the center of the platform and from there to the open arms.

### MWM test

The test was adapted from Morris et al. (21). The apparatus consists of a circular water pool 150 cm in diameter containing water to a depth of 8 cm. In the acquisition phase (D2 to D6), the mouse was required to find a hidden platform, positioned in the same place in all trials of this phase (the platform was submerged 2 cm below the water surface). Each mouse underwent four acquisition trials per day on five consecutive days. A trial was initiated by lowering the mouse into the water by its tail while facing the side wall of the pool at a predetermined position. The trial ended when the mouse found the platform or after 120 s had elapsed. If the mouse failed to find the platform within 120 s, it was gently guided to the platform and was allowed to remain there for 30 s. At the end of each trial, the mouse was dried and kept warm in a heated box for 15 min before the next trial. The results are reported as the mean of the four trials per day. The means obtained on the 5 consecutive days allowed us to define a learning curve (acquisition of spatial reference memory). One day after the acquisition phase (D7), the mice underwent the probe trial to assess memory retrieval. The hidden platform was removed from the water pool and the mice were allowed to swim for 60 s. The time spent and distance moved in the platform quadrant, and the number of crossings that coincided with the exact position of the platform, were recorded.

Because the MWM test was performed twice (before and after chronic intermittent exposure to ayahuasca), in the second test we decided to perform two probe trials: one before and another after the last acquisition phase. Consequently, a total of three probe trials was included in this study: probe trial 1: before chronic treatment, 1 day after the acquisition phase of the test; probe trial 2: after chronic treatment, 1 day before the acquisition phase of the test; and probe trial 3: after chronic treatment and after the acquisition phase of the test.

### Statistical analyses

Quantitative data are reported as means±SE. One-way ANOVA for repeated measures was used to analyze the results for 1) weight gain, 2) total locomotion (locomotor activity over 30 min) before and after exposure to ayahuasca, 3) locomotor habituation over 25 min (five blocks of 5 min each) either before or after treatment, 4) the acquisition phase of the MWM test (latency in finding the hidden platform during the 5 days), and 5) the time spent and distance moved, as well as the number of crossings over the platform position during the probe trials. The EPM parameters (percentage of entries and time spent in the open arms, number of entries into the closed arms and time in risk assessment) were analyzed by two independent one-way ANOVA tests, one for each period (before and after treatment with ayahuasca). ANOVA was followed by the Newman-Keuls *post hoc* multiple comparisons test, when necessary. The level of significance was set at P<0.05. All data analyses were done using SPSS statistics 21 software (IBM Corp., USA).

## Results

### Chronic intermittent exposure to ayahuasca did not alter weight gain throughout aging

A significant difference related to age [F_(11,10)_=23.069; P<0.001] was found but not related to treatment [F_(1,20)_=3.344; P=0.082]. There was no interaction between factors [F_(11,10)_=1.002; P=0.444]. As expected, the Newman-Keuls *post hoc* test showed a significant increase in body weight during the 12 months of treatment. Treatment with ayahuasca did not affect the pattern of weight gain during this period. Body weight at the beginning and end of treatment was 26.5±0.7 and 28.5±0.6 g *vs* 28.5±0.5 and 30.1±0.3 g for the Control and Ayahuasca groups, respectively.

### Locomotor activity

**Figure 1.**
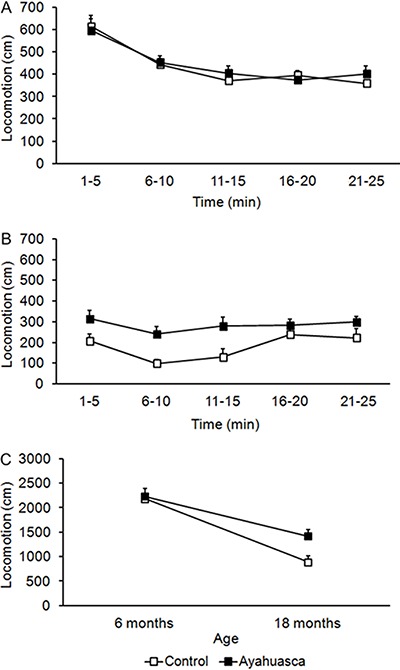
Locomotor activity in the open field test. *A*, Locomotor activity in five blocks of 5 min each, before treatment in 6-month-old mice. *B*, Locomotor activity in five blocks of 5 min each, after treatment in 18-month-old mice. *C*, Total locomotor activity (during 25 min) before and after treatment. Data are reported as the mean±SE (n=9 and 13, for the Control and Ayahuasca groups, respectively).

Figure 1 shows the locomotor activity (in five blocks of 5 min each) of mice before (6-month-old animals; panel *A*) and after (18-month-old animals; panel *B*) treatment with ayahuasca for 12 months, respectively. Panel *C* shows the total locomotor activity before and after treatment.

In 6-month-old mice, a significant difference over time was found [F_(4.17)_=28.114; P<0.001] but not between the control and treated groups [F_(1.20)_=0.028; P=0.868]. Furthermore, there was no interaction between factors [F_(4.17)_=1.340; P=0.262]. There was a significant decrease in the distance covered throughout the test that was detected early in the second block of 5 min in relation to the first block (P<0.001).

In 18-month-old mice, a difference over time [F_(4.17)_=4.662; P=0.002] and with treatment was found [F_(1.20)_=7.748; P=0.011], but there was no interaction between factors [F_(4.17)_=1.290, P=0.262]. The Newman-Keuls test showed that in the Control group there was a significant decrease (P<0.01) in locomotor activity in the second block of 5 min in relation to the fourth and fifth blocks.

Comparing before *vs* after ayahuasca treatment, a significant difference over time was found [F_(1.20)_=69.139; P<0.001], but not in relation to treatment [F_(1.20)_=3.118; P=0.093]; there was no interaction between factors [F_(1.20)_=4.82; P=0.57]. Locomotor activity decreased after treatment, regardless of the type of treatment (water or ayahuasca). In both groups, the locomotor activity was lower in 18-month-old mice compared to 6-month-old mice.

### EPM test

**Figure 2.**
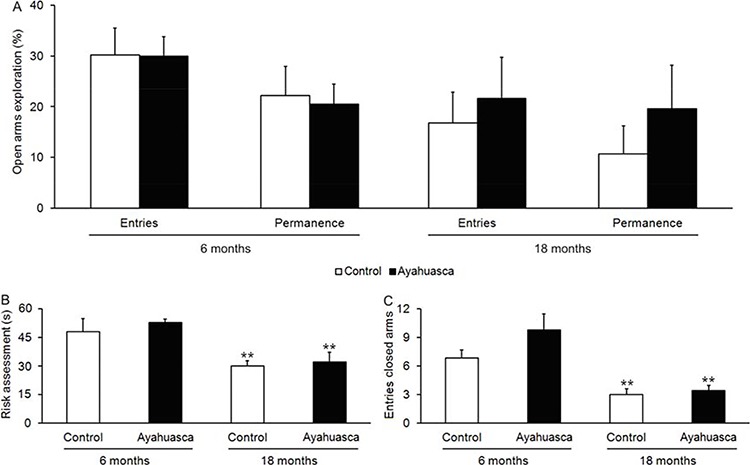
Behavioral measurements in the elevated plus maze (EPM) test before (6-month-old mice) and after (18-month-old mice) treatment with ayahuasca. *A*, Percentage of exploration in the open arms in relation to open and closed arms. *B*, Risk assessment behavior. *C*, Number of entries into the closed arm. Data are reported as the mean±SE (n=9 and 13, for the Control and Ayahuasca groups, respectively). **P<0.01 compared to the corresponding 6-month-old mice (ANOVA followed by Newman-Keuls *post hoc* test).

Figure 2 shows results for exploration in the open arms (*A*), risk assessment behavior (*B*) and the number of entries into the enclosed arms (*C*) before (6-month-old mice) and after treatment (18-month-old mice) with ayahuasca. In 6-month-old mice, one-way ANOVA showed no differences in the percentage of entries into [F_(1.20)_=0.001; P=0.973) and time spent in [F_(1.20)_=0.097; P=0.759] the open arms; there were also no differences in the number of entries into the closed arms [F_(1.20)_=1.948; P=0.178] and in the risk assessment behavior [F_(1.20)_=0.475; P=0.499].

In 18-month-old mice, one-way ANOVA showed no differences in the percentage of entries into [F_(1.20)_= 0.194; P=0.664) and time spent in [F_(1.20)_=0.633; P=0.436] the open arms; there were also no differences in the number of entries into the enclosed arms [F_(1.20)_=0.088; P=0.770] and in risk assessment behavior [F_(1.20)_=0.430; P=0.519].

### MWM test

**Figure 3.**
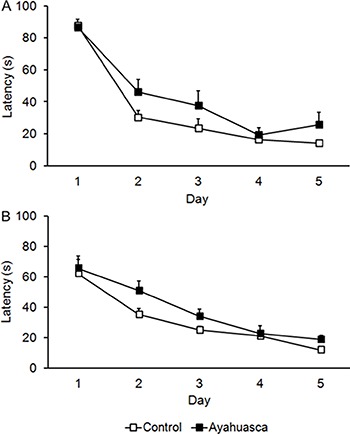
Latency during the acquisition phase of the Morris water maze (MWM) test. The latency to find the hidden platform was measured during five days of the acquisition phase. *A*, Before treatment (6-month-old mice). *B*, After treatment (18-month-old mice). Data are reported as the mean±SE (n=9 and 13, for the Control and Ayahuasca groups, respectively).

*Acquisition phase*. Figure 3 shows the latencies of the mice in finding the hidden platform during the 5 days of the acquisition phase before (6-month-old mice; panel A) and after (18-month-old mice; panel B) treatment with ayahuasca.

In 6-month-old mice, differences in relation to the test days were found [F_(4.17)_=51.411; P<0.001], but not between groups [F_(1.20)_=1.86; P=0.289]; there was no interaction between factors [F_(4.17)_=0.669; P=0.616]. The Newman-Keuls test revealed a significant decrease in the latency for finding the platform throughout the acquisition phase. This decrease was observed early in the second day of acquisition (P<0.01 in relation to the first day) and leveled off on the fourth day of acquisition (Figure 3A).

In 18-month-old mice, differences in relation to the test days were found [F_(4.17)_=3.429; P<0.001], but not in relation to treatment [F_(1.21)_=2.310; P=0.144]; there was no interaction between factors [F_(4.17 )_=0.687; P=0.603]. Regardless of the treatment, there was a progressive decrease in the latency throughout the days. The latency on the third day was lower than on the first day (P<0.01) and higher than on the fifth day (P<0.01; Figure 3B).

**Figure 4.**
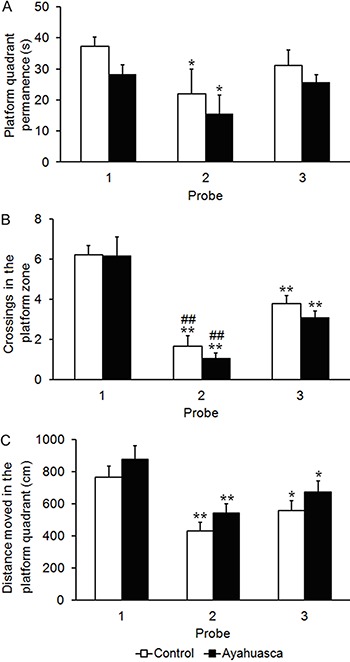
Behavioral measurements during probe trials in the Morris water maze (MWM) test. *A*, Time spent in the platform quadrant. *B*, Number of crossings in the platform zone. *C*, Distance moved in the platform quadrant. Data are reported as the mean±SE (n=9 and 13, for the Control and Ayahuasca groups, respectively). *P<0.05 and **P<0.01 compared to the first probe trial. ^# #^P<0.01 compared to the third probe trial (ANOVA for repeated measures followed by Newman-Keuls *post hoc* test).

*Probe trials.* Figure 4 shows the results of the probe trials. In relation to the time spent in the platform quadrant (Figure 4A), differences in the probe [F_(2.19)_=10.986; P<0.001] and treatment [F_(1.21)_=7.247; P=0.014] factors were detected. However, there was no interaction between factors [F_(2.19)_=0.2; P=0.82]. Regardless of the treatment condition, 18-month-old-mice spent more time in the platform quadrant in probe trial 3 compared to probe trial 2 (P<0.01).

In relation to the number of crossings in the platform quadrant (Figure 4B), differences in the probe were found [F_(2.19)_=30.739; P<0.001] but not for treatment factor [F_(1.21)_=0.622; P=0.425]; there was no interaction between factors [F_(2.19)_=0.15; P=0.861]. Regardless of the treatment condition, the number of crossings in probe 2 was lower than in probes 1 and 3 (P<0.001) and the number in probe 3 was lower than in probe 1 (P<0.01).

Finally, in relation to the distance moved in the platform quadrant (Figure 4C), differences were detected in relation to the probe [F_(2.19)_=13.935; P<0.001] but not the treatment [F_(1.21)_=3.285; P=0.085] factors; there was no interaction between factors [F_(2.19)_=0.001; P=0.999]. Regardless of the treatment, the distance traveled in the platform quadrant in probes 2 and 3 was shorter than in probe 1 (P<0.01); there was no difference between probes 2 and 3.

These results indicate that, regardless of the treatment condition, spatial reference memory acquired by 6-month-old mice did not persist to the age of 18 months. In addition, 18-month-old mice were able to express new spatial reference memory.

## Discussion

The results of this study indicate impairment in the evocation (but not acquisition) of spatial reference memory and in habituation to an open field during aging. However, this memory impairment was similar in the control and ayahuasca-treated groups. Moreover, neither age nor treatments altered the anxiety parameters analyzed in the EPM test. These findings indicate that chronic intermittent exposure to ayahuasca during aging neither impairs nor improves memory in mice.

Ayahuasca is a common beverage obtained from *B. caapi* and *P. viridis*. DMT from the leaves of *P. viridis* is a potent serotoninergic agonist, particularly at 5-HT_1A_, 5-HT_2A_ and 5-HT_2C_ receptors (3,4). The activation of these serotoninergic receptors can explain the hallucinogenic effects of ayahuasca. In contrast, the liana *B. caapi* contains the β-carbolines harmine, harmaline and tetrahydroharmine that are potent inhibitors of peripheral MAO-A, and MAO-A inhibition increases DMT bioavailability in the central nervous system (2).

Serotonin plays an important modulatory role in learning and memory (22). However, contradictory findings have been reported for the role of serotoninergic receptors in learning and memory, particularly for 5-HT_2A/2C_ and 5-HT_3_ receptors. For example, the inhibition or pharmacological blockade of serotonergic pathways has yielded divergent results for spatial memory tasks (23,24). In addition, 5-HT_1A_ receptors are abundantly expressed in the prefrontal cortex, amygdala and hippocampus and modulate serotonin actions related to fear, anxiety, stress and cognition (25). In a study of eight healthy volunteers, the 5-HT_1A/2A_ agonist psilocybin from *Psilocybe* mushrooms was found to selectively impair attentional tracking but not spatial working memory performance (26). Since DMT, a constituent of ayahuasca, is a potent agonist of 5-HT_1A_, 5-HT_2A_ and 5-HT_2C_ receptors, changes related to anxiety, learning and memory would be expected. For this reason, we assessed spatial learning and memory by examining a hippocampal-dependent visuospatial learning task in the MWM test and found no difference between control and ayahuasca-treated mice. There were also no changes in the anxiety parameters in the EPM, as reflected in the percentage of exploration in the open arms.

A neuroimaging study using single photon emission computerized tomography in 15 volunteers found that ayahuasca increased regional brain blood flow in the frontal and paralimbic areas. Additional increases were observed in the left amygdala/parahippocampal gyrus, a structure involved in emotionality (27). However, a longitudinal study of long-term ayahuasca use found no evidence of pathological alterations in mental health (personality, psychopathology, neuropsychology, life attitudes and psychosocial wellbeing), including in the Frontal Systems Behavior Scales in volunteers of two religious communities (28). The divergence between structural and behavioral studies may be partly explained by the pattern of ayahuasca use in religious rituals (ayahuasca is consumed a minimum of twice a month or more, but not daily) (2,8). Furthermore, the oral ingestion of ayahuasca reduces the bioavailability of DMT and other alkaloids present in the beverage.

Since the aim of this study was to mimic the religious pattern of ayahuasca consumption, the mice received ayahuasca orally twice a week. In addition, the ayahuasca treatment was maintained for one year since human consumption occurs for at least 10 years or throughout life (15). Interestingly, the long-term consumption of ayahuasca within a religious context does not appear to exert a deleterious effect on neuropsychological function (8,9). The results of the present study therefore agree with previous findings regarding the lack of deleterious behavioral effects of regular ayahuasca use on learning and memory. Indeed, aged mice performed worse than adult mice in both the water maze and open field tests, although the type of treatment did not influence this cognitive impairment.

Oxidative stress is involved in age-related cognitive impairment. The hippocampus plays a crucial role in certain forms of learning and memory and is thus particularly vulnerable to aging (13). Ayahuasca compounds have been reported to exert a neuroprotective effect. Preclinical studies have demonstrated a neuroprotective effect of harmine. In rats submitted to cerebral ischemia, harmine attenuated the cerebral infarct volume and reduced neuronal death. These effects could be partly attributed to the upregulation of glutamate transporter type 1 (29). Furthermore, a recent review of imaging studies in humans found that ayahuasca caused significant activation of brain nuclei, such as the frontal cortex and parahippocampal zone, which are related to learning and memory (30). Consequently, exposure to ayahuasca would be expected to prevent age-related cognitive impairment. However, as shown here, although there was significant impairment in the water maze and open field habituation in aged mice, the type of treatment (water or ayahuasca) did not influence the responses. These findings indicate that the neuroprotective effects cited in previous studies were unable to prevent aged-related cognitive impairment in the present study. This lack of correlation could partly reflect the pattern of ayahuasca treatment used in our animals (chronic intermittent exposure).

In conclusion, although ayahuasca modulates the monoaminergic system and can alter emotional and cognitive aspects, we have shown that chronic intermittent exposure to a dose of 1.5 mL/kg during aging did not change anxiety, learning and memory in mice. This conclusion is particularly relevant since our results were obtained using a schedule of treatment with ayahuasca that mimicked the pattern of beverage consumption in ritualistic contexts.
